# Three‐Dimensional Humanized Model of the Periodontal Gingival Pocket to Study Oral Microbiome

**DOI:** 10.1002/advs.202205473

**Published:** 2023-02-24

**Authors:** Miryam Adelfio, Zaira Martin‐Moldes, Joshua Erndt‐Marino, Lorenzo Tozzi, Margaret J. Duncan, Hatice Hasturk, David L. Kaplan, Chiara E. Ghezzi

**Affiliations:** ^1^ Department of Biomedical Engineering University of Massachusetts Lowell Lowell MA 01854 USA; ^2^ Department of Biomedical Engineering Tufts University Medford MA 02155 USA; ^3^ Department of Microbiology The Forsyth Institute Cambridge MA 02142 USA; ^4^ Center for Clinical and Translational Research The Forsyth Institute Cambridge MA 02142 USA

**Keywords:** host‐pathogen interactions, microbiome, nanomanufacturing, silk, tissue model

## Abstract

The oral cavity contains distinct microenvironments that serve as oral barriers, such as the non‐shedding surface of the teeth (e.g., enamel), the epithelial mucosa and gingival tissue (attached gingiva) where microbial communities coexist. The interactions and balances between these communities are responsible for oral tissue homeostasis or dysbiosis, that ultimately dictate health or disease. Disruption of this equilibrium can lead to chronic inflammation and permanent tissue damage in the case of chronic periodontitis. There are currently no experimental tissue models able to mimic the structural, physical, and metabolic conditions present in the human oral gingival tissue to support the long‐term investigation of host–pathogens imbalances. Herein, the authors report an in vitro 3D anatomical gingival tissue model, fabricated from silk biopolymer by casting a replica mold of an adult human mandibular gingiva to recreate a tooth‐gum unit. The model is based on human primary cultures that recapitulate physiological tissue organization, as well as a native oxygen gradient within the gingival pocket to support human subgingival plaque microbiome with a physiologically relevant level of microbial diversity up to 24 h. The modulation of inflammatory markers in the presence of oral microbiome indicates the humanized functional response of this model and establishes a new set of tools to investigate host–pathogen imbalances in gingivitis and periodontal diseases.

## Graphical abstract

The authors report an in vitro 3D anatomical gingival tissue model, based on silk and collagen proteins. The model recapitulates physiological tissue organization and a native oxygen gradient to support human oral human microbiome with a physiologically relevant microbial diversity. The modulation of inflammatory markers indicates the humanized functional response of this model, as a tool to investigate host–pathogen interactions.

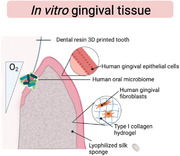

## 1 Introduction

The dynamic and polymicrobial oral microbiome is the initiator of diseases such as periodontitis and dental caries, globally two of the most predominant microbially induced disorders.^[^ ^1^ ^]^ The interactions and balances between the oral barriers and microbial communities are responsible for oral tissue homeostasis or dysbiosis, that ultimately dictate health or diseased tissue states, respectively. Disruption of this equilibrium is the first necessary step that ultimately leads to chronic inflammation and permanent tissue damage in the case of periodontitis.^[^ ^2^ ^]^ Due to mild initial symptoms as well as the large variety of clinical manifestations, the understanding of disease initiation and progression is difficult to establish.^[^ ^3^ ^]^ In fact, the current working polymicrobial synergy and dysbiosis model suggests that the disease is not originated by individual causative periodontopathogens, but due to the continuous cyclic interactions between physically and metabolically integrated polymicrobial communities and an imbalanced host inflammatory response.^[^ ^3^, ^4^ ^]^ Thus, emphasis is placed on identifying investigative tools to systematically study these complex interactions, rather than scrutinizing individual pathogenic elements, as in conventional culture‐based approaches.^[^ ^5^ ^]^

Host–pathogen interactions have been mainly investigated in clinical studies and animal models, with limited in vitro studies. Considering individual variability and diversity shown in clinical investigations,^[^ ^6^ ^]^ animal and in vitro models have offered advantages in elucidating the trajectory of disease. Humanized mice provide significant clinical observations, via mechanically or bacterial inoculum‐induced periodontitis models;^[^ ^7^ ^]^ however, they do not reflect the complex pathological scenario in the human condition due to differences in microbiome composition and progression of dysbiosis (i.e., animal model ligature of induced periodontitis).^[^ ^8^ ^]^ Current in vitro strategies are limited to 2D culture systems based on immortalized cells that are only functional for a short window of time (maximum 7 days).^[^ ^9^ ^]^ Such systems have been used to test irritant responses of new dental materials, dentifrices and oral care consumer products, but are unable to maintain the complexity of the oral pathogen community organization, due to the lack of the native oxygen and metabolic conditions. Moreover, current experimental models to study host–pathogen interplays rely on the use of planktonic bacteria cultures that neglect interspecies interactions required for attachment, colonization, and regulation of host mucosal communications and for diffusion of virulent soluble factors during disease onset and progression.^[^ ^4b^ ^]^ Indeed, plaque topography suggests that nutrient, moisture, and oxygen gradients promote the formation of microenvironments in which the oral microbiome actively maintains healthy homeostasis.^[^ ^10^ ^]^ Thus, there is a growing need to develop experimental tools to recreate the complex physical and metabolic conditions in the oral cavity, including oxygen and pH gradients, to accurately study host–pathogen interactions in vitro.

The reinvention of structural biopolymers as technical materials has enabled the use of natural polymers in applications that include drug delivery, regenerative medicine, and biosensing. In this context, the advanced nanomanufacturing of structural proteins such as collagen and silk into complex 3D architectures ranging from nano to macro scales has resulted in the production of unprecedented materials that can bridge the biotic/abiotic interface. Silk fibroin has been used as sutures for decades and has been recently cleared for new medical devices by the Food and Drug Administration approval (e.g., SilkVoice, Sofregen Inc.). Unlike other natural polymers, silks are uniquely high molecular weight, amphiphilic proteins that possess remarkable mechanical strength and toughness exceeding other commonly used degradable polymeric biomaterials.^[^ ^11^ ^]^ Silk regenerated from aqueous solution can form hydrogels, fibers, sponges and films with properties tailored to mimic tissue functions,^[^ ^12^ ^]^ with excellent biological performance both in vitro as well as in vivo.^[^ ^13^ ^]^ These technological and biological applications for silk as scaffolding materials support its use to generate a three dimensional (3D) tissue models in vitro. Such scaffolds provide biocompatibility, porous features for transport, robust and tunable mechanical properties, and retain their size and open porous structures for extended time due to slow proteolytic biodegradation, without a requirement for chemical crosslinking.^[^ ^14^ ^]^

**Figure 1:**
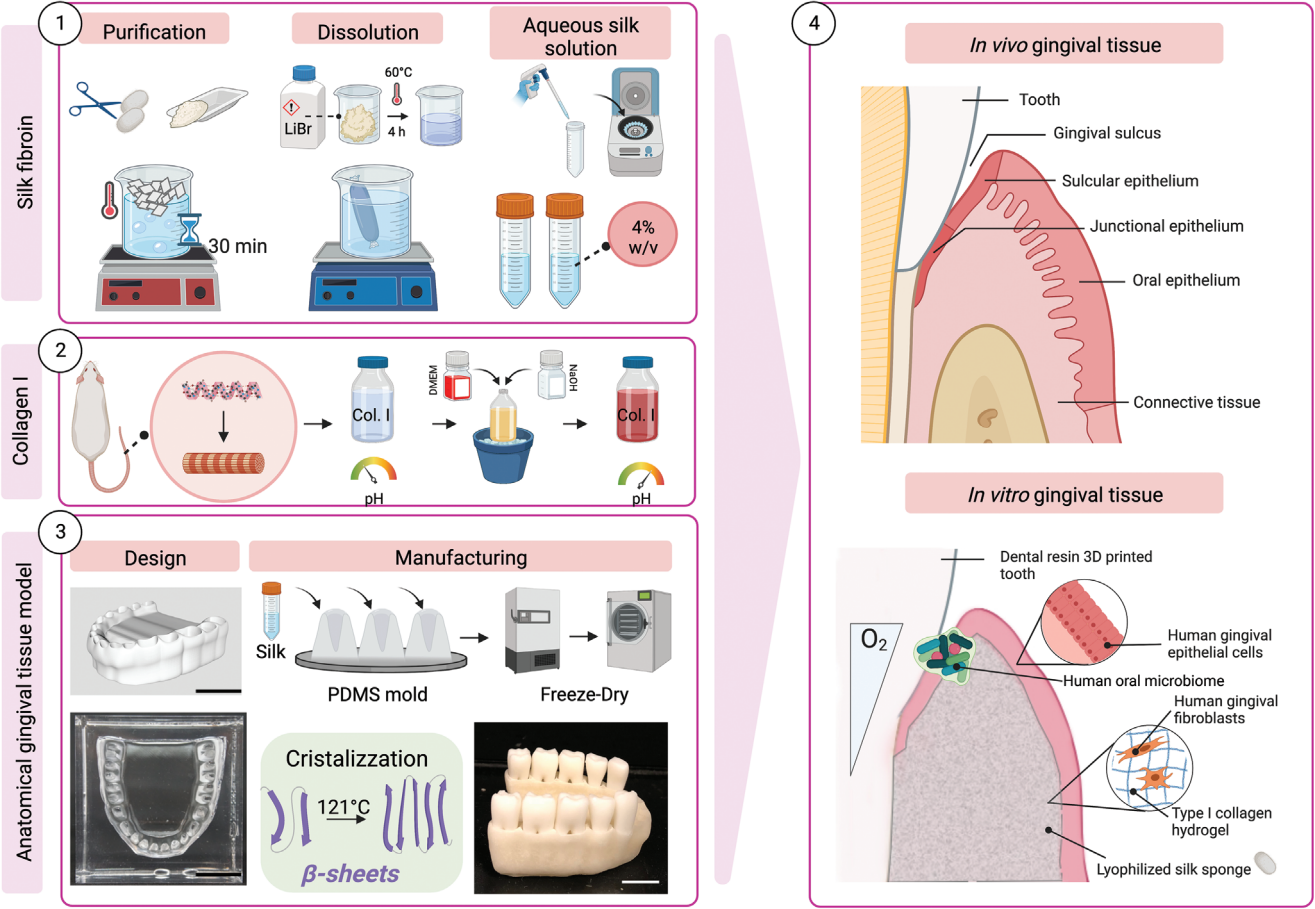
Experimental roadmap for the fabrication of the anatomical gingival tissue model. The workflow comprised of four steps. A) Silk proteins: silk solutions were obtained from *B. mori* silkworm cocoons, cut into small pieces, and boiled for 30 min; the fibers were dissolved, and the resulting solution was dialyzed against water and centrifuged to remove impurities. The final solution was then stored at 4 °C before use. B) Type I Collagen: type I Collagen isolated from rat tail was prepared by mixing DMEM and collagen solution and then neutralized by NaOH before use. C) Anatomical gingival tissue model: a SolidWorks rendering of a human gingival structure was generated to recreate a subsequent PDMS mold into which to cast the silk gingival scaffold (scale bar = 1 mm); the silk solution was pipetted into the PDMS mold and freeze‐dry to obtain a porous structure; subsequently, the scaffold was autoclaved to induce changes in the protein secondary structures, and 3D printed dental resin teeth were inserted into the scaffold to create the periodontal pocket. D) Overall approach: the complete construct was characterized by combining the three elements described above to mimic the in vivo features of gingival tissue. Briefly, human gingival fibroblasts were embedded in collagen I and seeded into the scaffold to replicate connective tissue; next, human gingival epithelial cells were seeded on the apical portion of the scaffold to replicate epithelial tissue. Finally, dental resin teeth were implanted to recreate the periodontal pocket in which the healthy microbiome, isolated from subgingival plaque, was inoculated to initiate host–pathogen interactions in vitro. Created with BioRender.com.

We recapitulated several in vivo oral features, including the native architecture, oxygen gradients and human‐derived multicellular populations, in an effort to provide a cost effective, robust and reliable experimental tool for acute longitudinal perturbation studies.^[^ ^15^ ^]^ Based on structural biopolymers as building blocks, we developed a humanized 3D gingival tissue model (**Figure** **1**) that recreates the native periodontal pocket and mimics the physiological oxygen tension, thus, able to support human microbiome persistence with a physiologically relevant level of microbial diversity. The modulation of inflammatory markers in the presence of a healthy oral microbiome supported the humanized functional response with this model. We anticipate that these efforts will open the door to future studies to elucidate initial interactions and balances between these two communities that are responsible for oral tissue homeostasis or dysbiosis, that ultimately dictates the healthy or diseased tissue state.

## 2 Results and Discussion

### 2.1 A Physiologically Relevant Tissue Model

**Figure 2:**
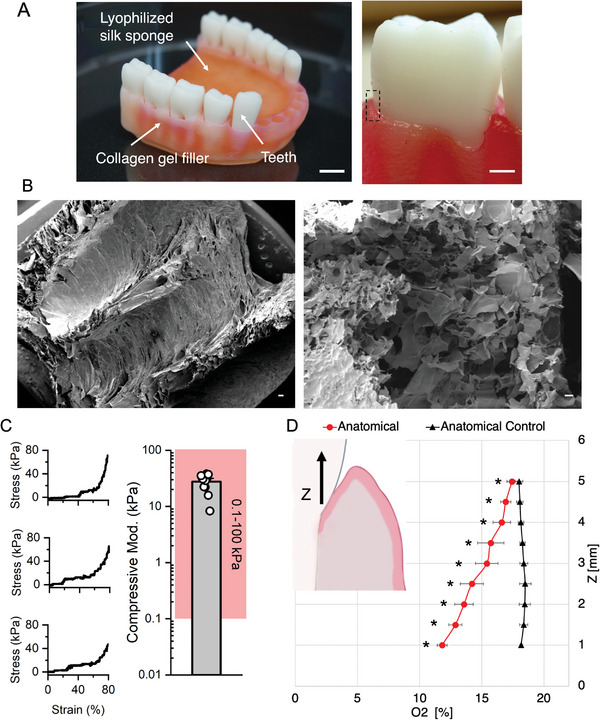
Structural, mechanical, and physical characterizations of the anatomical gingival tissue model. A) Macro image of the lower gingiva anatomical model with focus on the gingival sulcus where the teeth are inserted during the culture to recreate the gingival sulcus microenvironment. Scale bars = 3 mm. B) SEM micrographs of the lyophilized silk scaffold with details of the gingival sulcus (closed porosity) and connective structure (open porosity). Scale bars = 200 and 30 µm. C) Representative stress/strain curves of unconfined compression mechanical testing and compressive modulus (red area—range of mammalian soft tissue moduli) *n* = 6 for 3 independent experiments.^[^ ^19^ ^]^ D) Spatial oxygen profile of the cellularized anatomical model (red line) in comparison to the acellular model (black line), *n* = 5, two‐samples Student's *t*‐test, *p < 0.05). Data are plotted as mean ± SD.

Given the importance of a 3D structure in the gingiva architecture (i.e., epithelium and connective tissue interplay, mechanotransduction, and complex metabolism) and the lack of appropriate experimental tools, we aimed to develop a model that is physiologically relevant and facilitates the investigation of the large‐scale perturbations shown in oral disease, while being cost‐effective, robust and reliable. To successfully mimic the architecture of the gum, we developed an in vitro humanized gingiva, based on a silk protein porous scaffold to support the growth and persistence of native gingival multi‐cellular and microbial populations (Figure 1). The gingival scaffold was fabricated by casting a replica mold of an adult human mandibular (lower jaw) gingiva to recreate a tooth‐gum unit with a porous structure to support oxygen diffusion and nutrient distribution (**Figure** **2**). Teeth were 3D printed with dental resin, shown to be compatible with bacterial, adherence, viability and growth.^[^ ^16^ ^]^ The architecture of the scaffold was optimized to mirror healthy depth (0.69 mm) human dentogingival junction (sulcus) (Figure 2A,B),^[^ ^17^ ^]^ the space that lies between the gingiva and the tooth. Its increase in depth (≥4 mm) in more than two interproximal sites is clinical indication of periodontal disease.^[^ ^18^ ^]^ In addition, scaffold manufacturing parameters allowed the tuning of the compressive mechanical behavior of the lyophilized silk sponge to match the one of mammalian soft tissues (Figure 2C).^[^ ^19^ ^]^ To the best of our knowledge, this is the first gingival in vitro tissue model that recreates with high‐fidelity the anatomical niche where the oral host and microbiome exist synergistically. Previous studies have explored microbiome persistence on the apical surface of a Transwell system,^[^ ^20^ ^]^ on hydroxyapatite discs^[^ ^9c^ ^]^ or inserted into microfluidic devices^[^ ^21^ ^]^ to create direct contact with the underlying keratinocyte layer. These models replicated some of the features of the oral tissue (i.e., oral mucosa, cytokine secretion), they lacked the native pocket environment, and, more importantly, they neglected a normoxic to hypoxic gradient, which influences the eubiotic organization of polymicrobial communities and ultimately their crosstalk with the host cells and tissue. Moreover, clinical studies have correlated dysbiosis with increased depth (greater than 4 mm), hypoxic (O_2_: 1–3%), and acidic pockets in periodontal disease.^[^ ^18^, ^22^ ^]^ In our tissue model, the anatomical geometry of the sulcus creates a normoxic milieu at the epithelium–aerobic population interface, while providing a hypoxic niche for facultative and strictly anaerobic bacteria in the deep region of the pocket. To prove the presence of an oxygen gradient, we compared the performance of the anatomical tissue model to a planar silk sponge control, both populated with human primary oral cells (epithelial and stromal) (see Section 2.2); we then compared the physical characterization with the corresponding acellular constructs (planar and anatomical controls) (Figure 2D). The planar tissue construct was designed to have a similar geometry to that of a Transwell culture system and a stable oxygen profile along the *x*‐axis (Figure S1A, Supporting Information). Measurements in cellular anatomical sponges revealed an oxygen gradient with values comparable to the physiological range,^[^ ^23^ ^]^ with 18 ± 3 mmHg in the outer portion of the pocket and 11 ± 2 mmHg in the deep pocket (Figure 2D). Previous planar models have recorded a loss of biofilm viability due to the lack of hypoxia gradients in their systems,^[^ ^20^ ^]^ while others have grown bacterial lines in separated aerobic and anerobic conditions, subsequently co‐cultured for a short window of time.^[^ ^9c^ ^]^ Our results demonstrated the formation of localized oxygen tension, as a result of the native geometry and structural properties of the gingival scaffold. Taken together, those features provide a suitable environment for subgingival microbial communities, thereby, promoting their abundance and diversity.

### 2.2 Three‐Dimensional Oral Mucosal Tissue Equivalent

**Figure 3:**
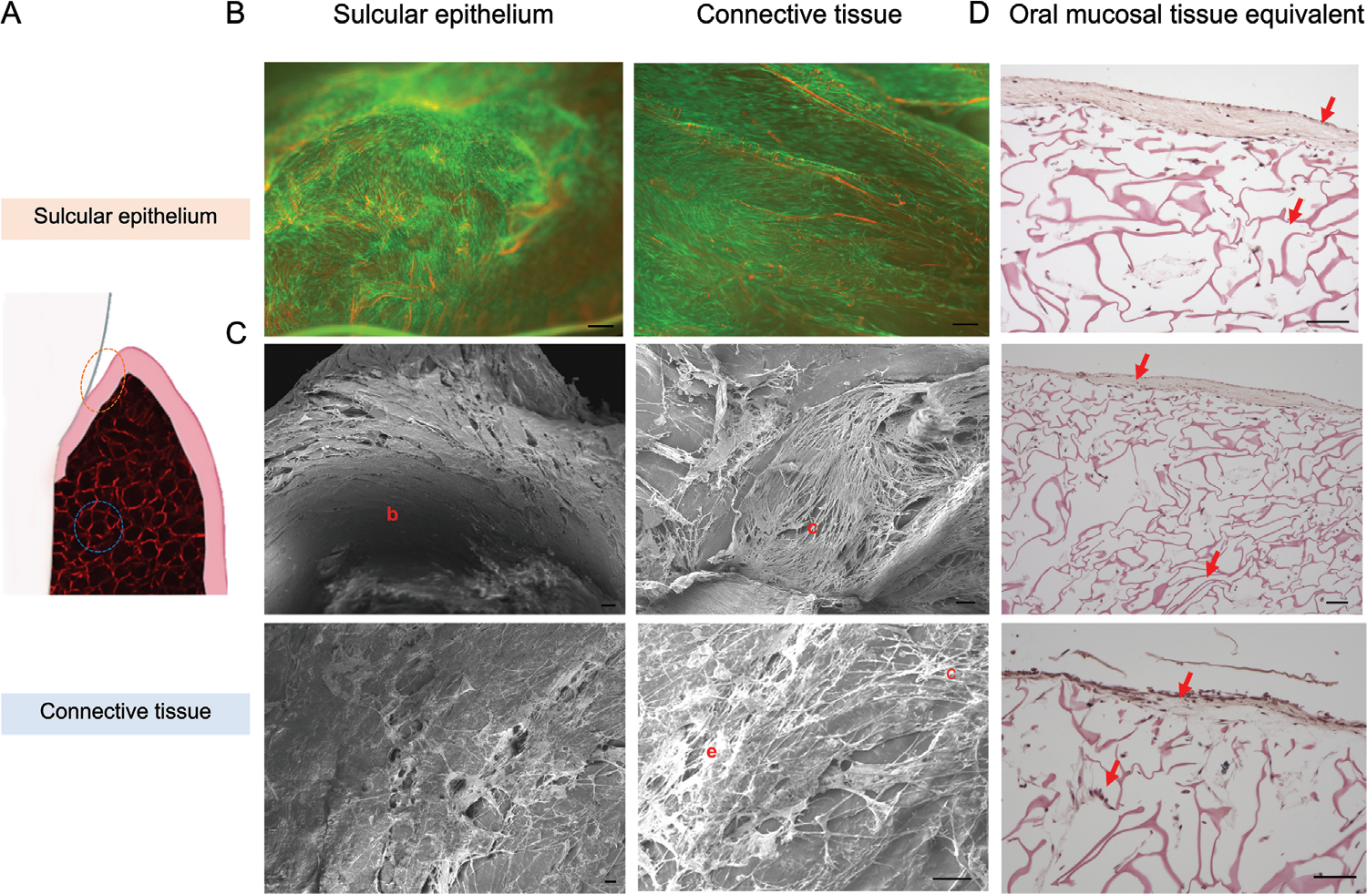
Biological assessments of the anatomical gingival tissue model. Sulcular epithelium and connective tissue viability and morphological characterization at 6 weeks in culture. A) Schematic of human gingival tissue showing epithelial and connective tissues, *n* = 5. Partially created in biorender.com. B) Maximum intensity projection of confocal laser scanning microscopy analysis of Calcein‐AM and Ki67 (Green and Red positive staining, respectively) labeled hGECs and hGFCs; C) SEM micrographs (basement membrane (b), connective tissue (c), and epithelial cells (e)). D) H&E staining of gingival anatomical model histological section, showing stratified sulcular epithelium and populated connective tissue. Red arrows indicate epithelium and connective tissues. *N* = 3. Scale bars = 100 µm.

**Figure 4:**
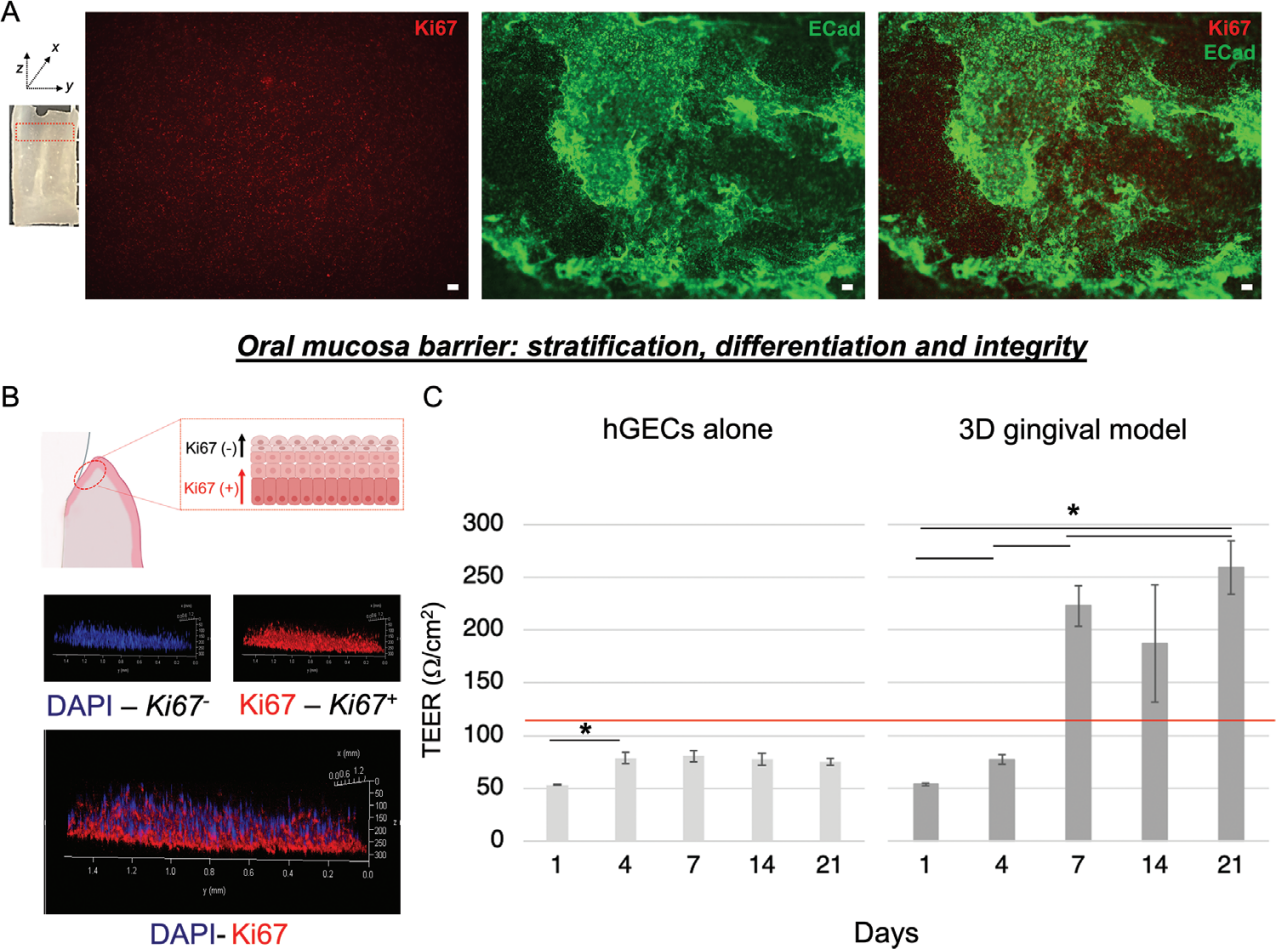
Epithelial barrier functional assessments. A) IHC assessment of epithelium proliferative profile and differentiation. Maximum intensity projection of confocal laser scanning microscopy analysis of Ki67 and ECad at 6 weeks in culture, *n* = 3. Scale bars = 100 µm. The red rectangular shape indicates the epithelial portion in the analyzed scaffold. B) Schematic of the epithelial barrier model and CLSM showing the proliferative and differentiated epithelium. C) Epithelial transmembrane resistance measured over time for anatomical gingival tissue model in comparison to hGECs alone and physiological gingival tissue reference (red line), *n* = 5 (one‐way ANOVA, with a Bonferroni and Tukey post hoc tests post hoc test for mean comparisons, *p < 0.05). Data are plotted as mean ± SD.^[^ ^60^ ^]^

Histologically, the gingiva is composed of oral mucosa, a barrier that separates the host from its environment and protects it against infections, but also connective tissue, that holds the gum firmly to the dentition.^[^ ^24^ ^]^ Considering the physiological importance of both tissues and their crosstalk, we aimed to replicate the gingival cytoarchitecture within the scaffold (Figure 1). To mimic the oral mucosa and submucosa, we employed human primary gingival (keratinocyte and stomal) cells; specifically, stromal cells were embedded in type I collagen extracellular matrix (ECM) to provide structural integrity inside the scaffold (Figure 1), while keratinocytes were seeded on the closed porosity of the scaffold. Previous gingival studies have used immortalized cell lines that can be expanded for prolonged passages, while partially recapitulating the histological features of the gum;^[^ ^9^, ^20^ ^]^ however, immortalized cells are limiting in mimicking cellular responses to *Porphyromonas gingivalis*, a keystone pathogen in periodontal disease.^[^ ^25^ ^]^ To assess cellular viability within the construct, primary gingival cells were labeled with Calcein‐AM dye, demonstrating prolonged viability up to six weeks in culture, based on confocal laser scanning microscopy analysis (CLSM) (**Figure** **3**A,B). These findings supported the ability of the scaffold to sustain the long‐term growth of human primary keratinocyte and stromal cells. The homogeneous distribution of the interconnected pores within the scaffold can maintain cell viability in the tissue constructs for extended periods favoring cellular growth, differentiation and native tissue functions, as previously shown in other in vitro studies conducted on intestinal,^[^ ^12c^ ^]^ brain^[^ ^26^ ^]^ or kidney^[^ ^27^ ^]^ tissues. Subsequently, to assess native histological features, we performed scanning electron microscopy (SEM) and histology imaging analyses (Figure 3C,D). SEM micrographs indicated the formation of a basement membrane characterized by stromal cells, which promoted the development of a multi‐layered epithelial structure above the stroma. Analysis of the upper sulcular epithelium showed the protrusion of lamellipodia in keratinocyte cells (Figure 3C higher magnification),^[^ ^28^ ^]^ while the remodeling of the connective tissue was demonstrated by new synthesis of collagen fibrils reorganized into individual and mesh‐like fibrils.^[^ ^29^ ^]^ Histological characterization of the anatomical model revealed the formation of the sulcular portion of the oral mucosa, a multilayered stratified epithelium tightly bounded by dense underlying connective tissue (Figure 3D), indicating an organization similar to the native gum tissue.^[^ ^30^ ^]^ Lastly, the epithelium displayed a physiological phenotypic appearance, as shown by the positive E‐Cadherin staining (**Figure** **4**A) and Figure S2, Supporting Information, a protein essential in the formation of adherent junctions between cells, to suggest the presence of epithelial barrier integrity.^[^ ^30a^ ^]^ Additionally, to highlight the multi‐layering and differentiation within the epithelium, we counter‐stained keratinocytes with Ki67, a protein that is not expressed in quiescent cells (G0 phase)^[^ ^31^ ^]^ and thus distinguishes cells in a state of active proliferation from differentiated (quiescent) cells forming the shedding epithelium^[^ ^20^ ^]^ (Figure 4A,B). Immunohistochemistry results indicated Ki67 positivity in the lower layer, demonstrating the gradient of proliferative cells within the multi‐layered epithelium, while strong expression of E‐Cadherin in both the lower and upper layers, supporting the epithelium barrier formation^[^ ^20^, ^32^ ^]^ (Figure 4A,B) and Figure S2, Supporting Information. To functionally characterize the barrier integrity of the anatomical gingival model, we quantified the Trans Electrical Epithelium Resistance (TEER) in a planar construct populated with gingival cells (Figure 4C). As an experimental control and to align with the current literature, we quantified the epithelial barrier activity in a 2D Transwell culture system of human primary gingival keratinocytes co‐cultured with human primary stromal cells. The anatomical gingival tissue model displayed an increase in TEER overtime, with significantly higher values in comparison to the Transwell co‐culture control. This confirmed the ability of the tissue model to support a stable functional epithelial barrier over extended time in culture. After seven days, the anatomical construct displayed stable TEER values within the physiological range of gingival epithelial multilayers^[^ ^33^ ^]^ for the entire duration of the culture. Taken together, these data corroborated the use of structural proteins, such as silk and type I collagen, as building material for the development of long‐term 3D tissue models. In addition, these findings demonstrated the successful integration and differentiation of human primary gingival cells within the anatomical tissue model,^[^ ^34^ ^]^ as well as the recapitulation of the oral mucosa and submucosa in vivo features.

### 2.3 In Vitro Maintenance of a Complex Human Oral Microbiome

**Figure 5:**
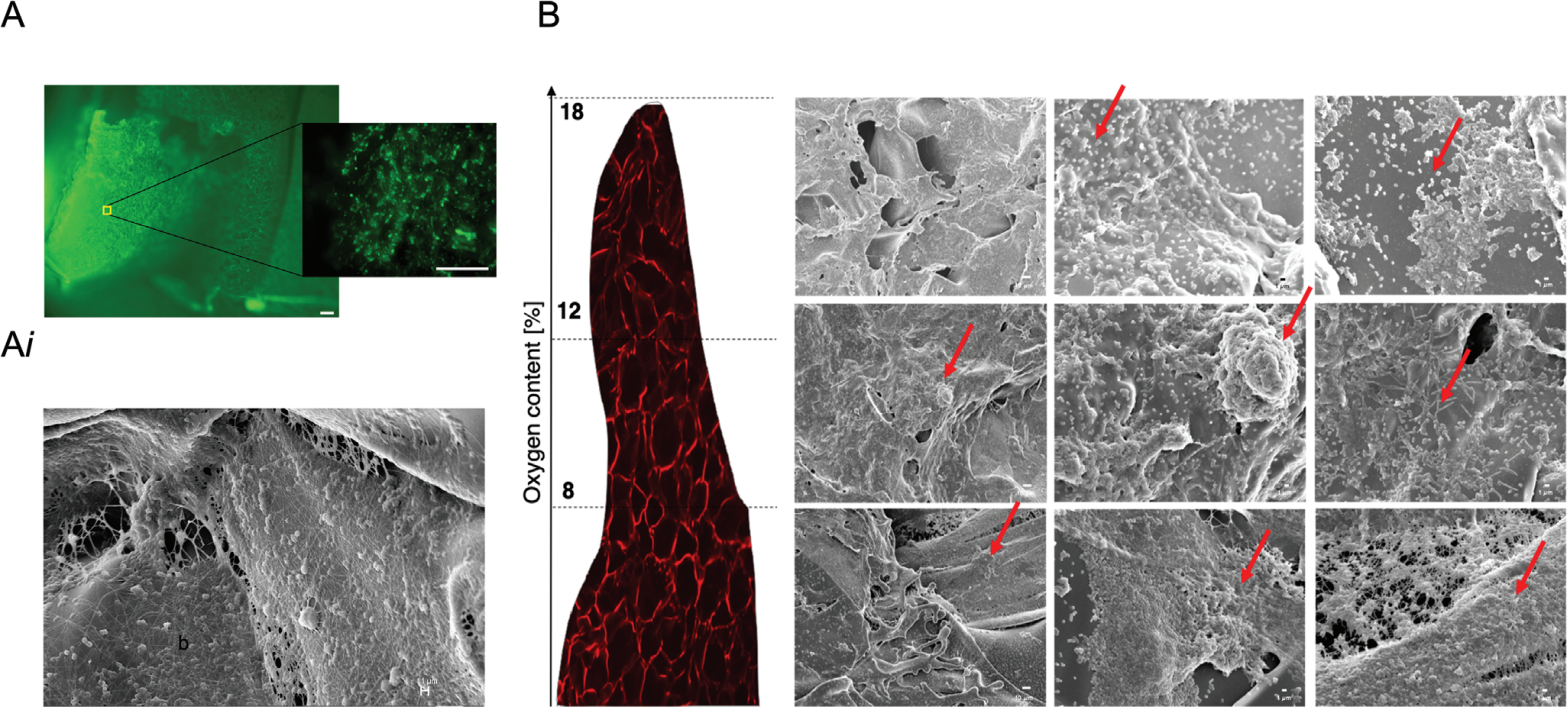
Human oral microbial viability and organization. A) Microbiome viability assessment at 24 h in the anatomical gingival tissue model. Maximum intensity projection of CLSM analysis of Syto‐9 positively stained human oral microbiome. Scale bars = 100 µm. A‐i) Scanning electron microscopy analysis showing biofilm (b) formation. B) Scanning electron microscopy analysis demonstrated a gradient of plaque organization as a function of the oxygen content. Red arrows indicate the growth and aggregation of the microbiome on the anatomical model. *N* = 5.

To replicate the physiological polymicrobial dynamics within the biofilm and initiate interactions with the host, we inoculated human subgingival plaque collected from healthy patients into the periodontal pocket (**Figure** **5**). Physiologically, the host provides a suitable ecosystem in which microbial communities can adhere, live, instruct the host immune response and suppress the initial growth of pathogenic species to maintain homeostasis.^[^ ^4^, ^35^ ^]^ To support tissue homeostasis in vitro, we supplemented the coculture media with pooled human saliva, in an effort to complement community nutrition and pH stabilization (i.e., urea, citrate, uric acid),^[^ ^36^ ^]^ but also to protect mammalian cells, as human saliva contains antimicrobial factors^[^ ^4^, ^35^ ^]^ (i.e., lysozyme or lactoferrin). Syto9 staining confirmed attachment and the integration of the microbial communities within the construct after 24 h (Figure 5A). To further characterize the microbiome in the anatomical model, we investigated biofilm distribution (Figure 5A‐i) as a result of the oxygen content, by imaging three regions of the construct that were previously identified through oxygen measurements. SEM micrographs (Figure 5B) showed a microbial presence, as a function of oxygen level, and aggregation into round‐shaped and corncob structures in the regions of the periodontal pocket, as an indication of biofilm formation. Data in literature indicated that microbiome biogeography dictates their ecology and physiology, as well as their interplay with the host.^[^ ^37^ ^]^ Additionally, corncob structures have been previously identified in subgingival plaque samples as an index of physical direct contact of different genera of bacteria for assemblage or attachment (i.e., *Streptococcus* and *Aggregatibacter*).^[^ ^37^ ^]^ We have shown that the oral microbiota was distributed along the periodontal pocket, potentially contributing to differences in microbial community composition in the different locations. Therefore, we were able to demonstrate that the anatomical architecture of the tissue model determined an oxygen gradient, that can instruct future studies on microbial biogeography, by investigating the spatial distribution of the biodiversity of oral bacteria. Given the qualitative assessments from SEM analysis, future studies will be focused on fluorescence in situ hybridization (FISH) and site‐specific sequencing analyses to correlate identification of bacterial taxa to their biogeography.

### 2.4 Microbial Identity in the Anatomical Gingival Tissue Model: 16S rRNA

**Figure 6:**
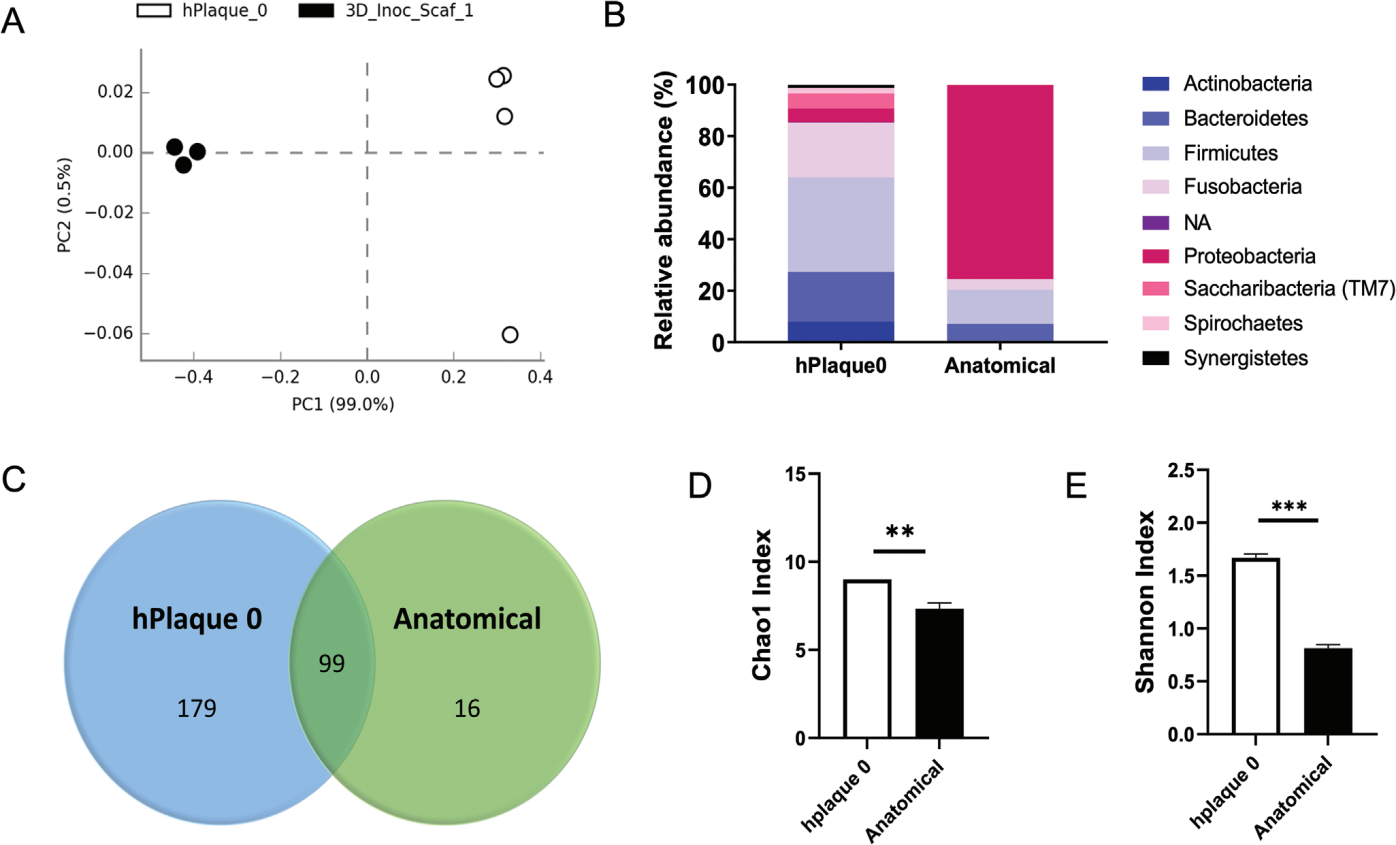
Analysis of the diversity and relative abundance of human oral microbiome in the anatomical gingival model. A) Principal component analysis (PCA) clustering of oral microbial populations generated after 16S rRNA sequencing of samples collected from human plaque (Initial Microbiome) and at 24 h in the anatomical model (24 h Microbiome). Each point represents one sample. B) Relative abundance distribution of main phyla detected in the Initial Microbiome samples and samples collected at 24 h in the anatomical model (Anatomical). C) Venn diagram showing unique and shared OTUs. D) Alpha diversity representation of oral microbiota. E) Richness representation of oral microbiota. Statistical analysis: ANOVA test with a *t*‐Student post hoc test. *p < 0.05; **p < 0.01; ***p < 0.001.

**Figure 7:**
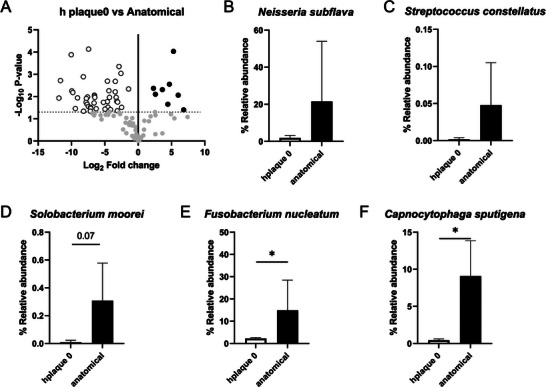
Ad‐hoc specie analysis of human oral microbiome in the anatomical gingival model. A) Volcano plot of specie level. The *x* axes display the fold changes in relative abundance (log2) between original oral plaque samples (Initial Microbiome) and at 24 h in the anatomical model (24 h Microbiome) data sets; the *y* axes display the log of the *p* values of the test statistic. The dashed horizontal line at log 1.3 corresponds to a *p* value of 0.05. Enrichment of the specie in the Initial Microbiome samples is represented as white circles, whereas enrichment of the specie in the anatomical model samples is represented with black circles. The full list of species showing significant shifts in relative abundance is provided in Table S1, Supporting Information. B–F) Relative abundance of *Neisseria subflava* (B), *Streptococcus constellatus* (C), *Solobacterium moorei* (*p* value = 0.07) (D), *Fusobacterium nucleatum* subsp. polymorphum (E), and *Capnocytophaga sputigena* (F). Statistical analysis: ANOVA test with a *t*‐student post hoc test. *p < 0.05.

Multiple species of bacteria inhabit the oral cavity and molecular studies have been conducted to identify the molecular bacterial signature and understand which genera are under or over‐represented in eubiotic or dysbiotic scenarios. Our experimental tissue model aims to mimic periodontal health. To analyze the ability of the anatomical tissue model to sustain the growth of a healthy oral microbiota, we performed a 16S rRNA sequencing analysis comparing the bacterial profile obtained after a one‐day incubation period (24 h Microbiome Anatomical Model) with the original sample (Initial Microbiome) (**Figure** **6**). Principal component analysis (PCA) (Figure 6A) showed a dissimilarity in the oral microbiota between the two conditions, confirmed by the relative abundance of the phyla of the microbial populations (Figure 6B). Compared with Initial Microbiome, we found that the anatomical tissue model supported the viability and growth of the complex human microbiota. Indeed, of the nine Operational Taxonomic Units (OTUs) identified in the Initial Microbiome as phyla, eight were retained in the anatomical model. In particular, we found that phylum *Proteobacteria*, which is associated with periodontal health,^[^ ^38^ ^]^ was highly enriched after one day of incubation in the anatomical model (Figure 6B), while some phyla were reduced, especially *Actinobacteria*, Saccharibacteria and Synergistetes, or even gone such as Spirochaetes (Figure 6B). Although we could not mimic the exact relative abundance found in the Initial Microbiome 1 day after inoculation, we were able to preserve the majority of the richness and diversity with aerobic and anaerobic species coexisting in the anatomical model. In fact, we were able to identify a total of 294 OTUs, including 99 OTUs shared among the samples (Figure 6C). Additionally, both the *α*‐diversity, as determined by Shannon index, and the richness, as determined by Chao‐1 index, showed significant statistical reduction from the original sample in the anatomical model (Figure 6D,E). Both reductions were associated with short‐term culture period of the tissue model. In fact, in vitro studies conducted on the gut microbiome have reported an initial reduction in richness and diversity that stabilizes after a prolonged period in culture.^[^ ^39^ ^]^ To further elaborate on OTUs retained in the anatomical model, we characterized the microbiome as genus in the taxonomic rank (Figure S3, Supporting Information). The analysis revealed a total of 82 genera, including *Streptococcus, Actinomyces, Fusobacterium, Neisseria, Porphyromonas, Prevotella, Capnocytophaga, Rothia, Leptotrichia* and *Veillonella*.^[^ ^40^, ^41^, ^42^ ^]^ Compared against the Initial Microbiome, a decrease in the relative abundance in the anatomical samples for *Actinomyces, Porphyromonas, Prevotella, Rothia, Leptotrichia* and *Veillonella*, while similar levels of relative abundance for *Fusobacterium*, and an increase in relative abundance of *Streptococcus, Neisseria* and *Capnocytophaga* in the anatomical samples was found. Interestingly, *Streptococcus* and *Fusobacterium* are among the major bacterial genera that colonize the oral cavity and form crucial constituents of dental plaque (i.e., dental biofilms accumulating on non‐shedding tooth surfaces). *Fusobacterium* members acts as a bridge between early (*Streptococcus*) and late colonizers, coaggregating with most oral bacteria.^[^ ^43^ ^]^ These findings correlate with the in vivo tissue‐specific tropisms mimicked in the anatomical tissue model, in which the presence of colonizers facilitates structural polymicrobial synergies and thus colonization and aggregation of other bacteria on oral surfaces.^[^ ^4b^ ^]^ Lastly, to further analyze changes in the relative abundance of the microbiota between the Initial Microbiome and the anatomical model, we generated a volcano plot and characterized the microbiome as species in the taxonomic rank. The volcano plot represents the fold change in the relative abundance; specifically, a negative fold change where less than −2 denotes species enrichment in the Initial Microbiome samples (white circles), whereas a positive fold change greater than 2 denotes species enrichment in the anatomical model samples (black circles) with a 95% confidence interval (**Figure** **7**A). A total of 50 species showed a statistical difference in relative abundance, 42 corresponding to the Initial Microbiome samples, and 8 corresponding to the anatomical model (Table S1, Supporting Information). The 8 species with the highest abundance were identified as members of the genus *Neisseria* and *Gemella*, a member of the order Lactobacillales, and the species *Neisseria subflava, Streptococcus constellatus, Solobacterium moorei, Fusobacterium nucleatum subsp. polymorphum* and *Capnocytophaga sputigena*. Interestingly, only *N. subflava* is considered aerobic, while the others are considered either facultative anaerobes or strict anaerobes, like *F. nucleatum subsp. polymorphum*. In comparison to the Initial Microbiome, we observed an increase in the relative abundance of all species in the anatomical samples, which in the case of *F. nucleatum subsp. polymorphum* and *Capnocytophaga sputigena* was statistically significant (Figure 7B,F). With regard to the statistical difference of some species between the two conditions, this could be associated with several factors, including saliva supplementation, directly affecting the composition of the subgingival microbiome (i.e., environmental factors, personal hygiene, sex, or periopathogens),^[^ ^44^ ^]^ as well as experimental conditions, such as components of co‐culture media or teeth for attachment. Altogether, these results demonstrated that the 3D anatomical gingival tissue model supported the survival and growth of key members of the healthy human oral microbiota (aerobic, facultative, and strictly anaerobic), including previously uncultivable bacteria (i.e., *Saccharibacteria*‐TM7).^[^ ^18^, ^45^ ^]^

### 2.5 Tissue Response to Human Oral Microbiome: Cytokine Profile

The gingival sulcus and periodontal pocket provide the commensal bacteria with a stable ecological niche and in return the oral microbiota locally supports host health by forming symbiotic biofilms that balance pH and suppress pathogen growth, under healthy conditions. In this context, the sulcular and junctional epithelia represent immunologically active sensors of the proximal plaque biofilm. They play a homeostatic role in the healthy periodontium, by generating a gradient of chemokines for the recruitment of immune cells to the gingival crevice, contributing to the maintenance of tissue homeostasis.^[^ ^46^ ^]^ The transition to a state of dysbiosis is regulated by several processes, including imbalanced interaction among bacteria, the presence of individual microbial species (pathobionts), a poorly controlled immune response and effect of diet and external conditions.^[^ ^4b^ ^]^ As a new community develops, microbial, and host‐immune response by‐products dictate changes to the local environment that further facilitate the outgrowth of microorganisms associated with a dysbiotic state. The subsequent tissue disruption leads to the over‐activation of the immune system, resulting in the chronic inflammatory state, fueled by the positive‐feedback loop of pro‐inflammatory cytokines secretion.^[^ ^46b^ ^]^

**Figure 8:**
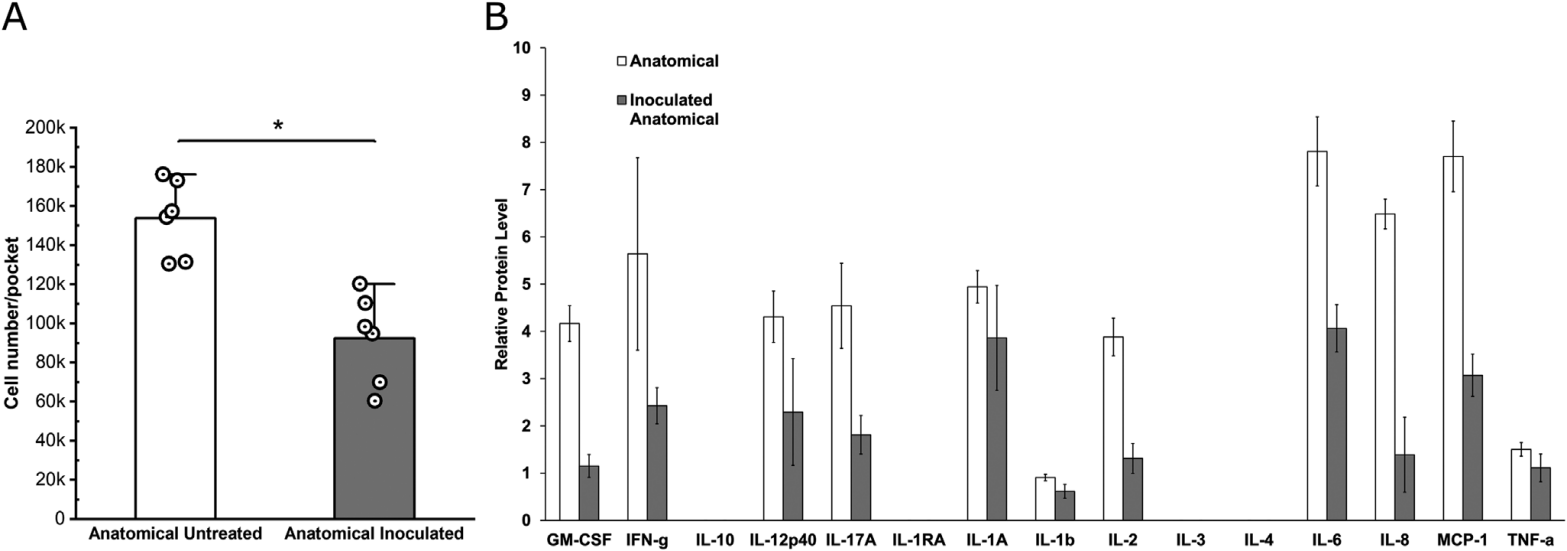
Tissue response to human oral microbiome: viability and cytokine profile. A) Cell viability within gingival sulcus was quantified via PicoGreen in the anatomical inoculated model in comparison to the untreated at 24 h post‐inoculation. *N* = 6, two‐samples Student's *t*‐test, *p < 0.05. B). Simultaneous analysis of multiple cytokine and chemokine biomarkers with Bead‐Based Multiplex Assays using the Luminex technology at 24 h upon inoculation from gingival sulcus exudates. *Significant effect of human microbiome interaction on the anatomical gingival tissue model (one‐way ANOVA, with a Bonferroni and Tukey post hoc tests post hoc test for mean comparisons, *p < 0.05). Data are shown as mean ± SD.

To validate the clinical relevance of the 3D gingival model, we investigated the initial response of the anatomical tissue model to the human microbiome inoculated from healthy patients. A reduction in cell viability was observed within the pocket, from DNA quantification analysis (**Figure** **8**A), in response to human microbiome exposure. In both samples, pro‐inflammatory (granulocyte‐macrophage colony‐stimulating factor [GM‐CSF], IL‐1RA, IL‐1*α*, IL‐1*β*, IL‐6, IL‐8, IL‐12p40, IL‐17A, and tumor Necrosis factor alpha [TNF‐*α*]) and anti‐inflammatory (IFN‐*γ*, IL‐2, IL‐10, IL‐3, and IL‐4) cytokines were above the detection limits, except for IL‐10, IL‐1RA, IL‐3 and IL‐4 (Figure 8B). In comparison to the untreated anatomical tissue model, the inoculated anatomical group showed a decrease of both pro‐inflammatory and anti‐inflammatory cytokines (Figure 8B). Cytokine downregulation has been previously reported as part of the initial response of the gingival epithelium to interactions with oral microbiome.^[^ ^46^, ^47^ ^]^ For example, IL‐1*β* and IL‐8 were statistically reduced in the presence of the microbiome in the anatomical tissue model compared to the untreated version (Figure 8B), as shown in in vivo findings, where moderate plaque formation is considered in the normal range of surveillance mechanisms by the host.^[^ ^46c^ ^]^ In contrast, increased cytokine release is associated with inflammatory gingival conditions.^[^ ^47^, ^48^ ^]^ Overall, the tissue model supported a functional response to human oral microbiome interactions in healthy conditions. It is important to note that the current model presents some limitations, that will be address in future studies, focused on extending the investigation window beyond 24 h with clinical relevance by increasing sample size. The model is currently lacking additional key elements of the human gingiva, such as bone, vessels, immune system elements (i.e., neutrophils and macrophages) and salivary flow, which are crucial to fully mimic the evolution of periodontal disease in vitro.

## 3 Conclusion

The aim of this study was to develop an in vitro humanized gingival model that mimics the in vivo anatomical and cytoarchitectural features of the gingiva to study initial host–microbiome interactions. It has been hypothesized that chronic periodontitis results from gingivitis progression;^[^ ^2^ ^]^ however, current technologies are limited and fail to sustain and recapitulate the complex host–pathogen interplay under healthy and disease conditions. To the best of our knowledge, no other oral tissue model has been able to mimic the gingival anatomical architecture and the physiological oxygen tension that, together with oral mucosa stratification, allow aggregation and organization of the microbiome while maintaining diversity in vitro. Overall, our results supported the establishment of a tissue phenotype, maintenance of an eubiotic microbiome after one day of culture, and inflammatory signaling that were calibrated with early clinical host–microbiome interactions. In conclusion, this anatomical humanized gingival tissue model should provide a robust and reliable experimental tool for perturbation studies. Thus, further development of this tissue model past 24 h would have the potential to open the door to the identification of predictive disease biomarkers within the oral microbiota and beyond, and should underpin the development of intervention strategies to promote overall human health.

## 4 Experimental Section

### Gingival Tissue Model Fabrication

The scaffolding process used a water‐based silk technology to create highly porous silk scaffolds for implants and tissue regeneration.^[^ ^19^ ^]^ Highly interconnected pore architectures were achieved in the silk scaffolds by freezing and lyophilizing the water in the silk fibroin aqueous solution.

Aqueous silk solution was prepared from *Bombyx mori* silkworm cocoons, following the experimental procedure described in previous studies.^[^ ^49^ ^]^ *B. mori* silk cocoons were purchased from Tajima Shoji Co. (Yokohama, Japan). Briefly, the cocoons were degummed by boiling in 0.02‐m sodium carbonate (Sigma Aldrich, St Louis, MO) solution for 30 min. The extracted fibroin was then rinsed three times in Milli‐Q water, dissolved in a 9.3‐m LiBr solution yielding a 20% (w/v) solution, and subsequently dialyzed (MWCO 3,500) against distilled water for 2 days to obtain silk fibroin aqueous solution at the approximate concentration of 8% (w/v), as determined by gravimetric analysis.

The silk anatomical gingival scaffolds were prepared using a replica molding technique (Figure 1). The design of a human adult gingival anatomical model and teeth were purchased from CadHuman.com and used to 3D print mandibular jaw gingiva structure with a Form2 printer in combination with a white dental resin (formlabs, Somerville, MA, USA). Sylgard 184 silicone elastomer kit was prepared in a 10:1 ratio, where the 3D printed model was inserted, and placed for 3 h in a 70 °C oven to cure.

Upon model retrieval, the anatomical mold was used to cast the aqueous silk solution (4 mL at 4% wt/v in deionized water). Dry scaffolds were removed from the molds and post‐processed by autoclaving at 121 °C for 20 min at 15 psi to induced *β*‐sheets formation, thus resulting in protein insolubility in aqueous environments. Scaffolds were rehydrated in deionized water. Control planar control scaffolds were similarly prepared by dispensing the aqueous silk solution into wells of standard 24‐well cell culture plates (1 mL/well) (Corning, NY), and the sliced with a micron cutter paired with a razor blade. All scaffolds were then sterilized by autoclave in deionized water before cell culture procedures.

Human gingival epithelial cells (hGECs) and human gingival fibroblast cells (hGFCs) were purchased from Lifeline Cell Technology (Frederick, MD), derived from the mandibular gingiva. Gingival cells were maintained in culture up to passage 6 in appropriate medium supplemented with associated growth factor kits (Lifeline Cell Technology, Frederick, MD). hGECs were confirmed positive for cytokeratins K13 and K14. Co‐culture conditions were optimized to support the growth and differentiation of both hGECs and hGFCs, by using three parts of basal FibroLife Serum‐Free Medium and one part of DermaLife Basal Medium, supplemented with the following growth factors: human serum albumin (500 µg mL^−1^), linoleic acid (0.6 µm), lecithin (0.6 µg mL^−1^), recombinant human fibroblasts growth factors (5 ng mL^−1^), recombinant human epidermal growth factor (5 ng mL^−1^), recombinant human transforming growth factor beta 1 (30 pg mL^−1^), recombinant human insulin (5 µg mL^−1^), ascorbic acid (50 µg mL^−1^), L‐glutamine (7.5 mm), hydrocortisone hemisuccinate (1 µg mL^−1^), epinephrine (1 µm), apo‐transferrin (5 µg mL^−1^), recombinant human transforming growth factor alpha (0.5 ng mL^−1^), gentamicin (30 µg mL^−1^), and amphotericin (15 ng mL^−1^).

The surface of the anatomical scaffolds was used to accommodate hGECs, while the porous bulk space housed hGFCs. hGFCs were seeded into the anatomical scaffold via neutralized rat tail collagen type I gel (First Link UK Ltd.) at 200 000 cells/mL to allow uniform distribution of the cell population. Collagen gel solution was prepared by mixing four parts of collagen type I and one part of Dulbecco's Modified Eagle's Medium (DMEM) 10× (Sigma Aldrich, St Louis, MO) and neutralized in sodium hydroxide 10 m (Sigma Aldrich, St Louis, MO). After gelation time (37 °C for 30 min), the construct was seeded with hGECs at a density of 50 000 cells/cm^2^. After 2 h in incubation at 37 °C, the construct was flipped and seeded on the other side with hGECs and incubated for another 2 h. During the incubation, a small amount of co‐culture medium would be dripped on the scaffolds to keep them moist. The cellularized constructs were then incubated for 1 week submerged in co‐culture media until the hGECs reached confluence. Dental resin teeth were inserted in the gingival sulcus after a week post‐seeding and the level of media was adjusted so that hGECs close to the gingival sulcus were exposed to air liquid interface (ALI) to induce differentiation, while maintaining the base of the sponge in media. The gingival model was maintained in a humidified incubator at 37 °C with 5% CO_2_. Culture media was changed every day for the entire duration of the experiments. Planar silk sponge constructs were used as controls and prepared as described above. hGFCs in neutralized collagen type I were seeded on the open porosity of the scaffold to recreate the bulk of the tissue. After gelation, the tissue model was flipped and accommodated inside a Trans‐well insert (Corning, NY) and hGECs were seeded on the closed porosity surface. Culture conditions were the same as above.

### Gingival Tissue Model Characterization

The morphology and distribution of the pores, cellular morphology and organization, and microbiome distribution within the tissue model were characterized by SEM. The constructs were harvested, and teeth were removed prior to the fixation process. After rinsing in phosphate buffered saline (PBS) (1×) (Thermo‐Fisher Scientific, Waltham, MA), the constructs were fixed in 4% paraformaldehyde‐0.1 m sodium cacodylate solution overnight at 4 °C. After washing with deionized distilled water, the pockets were cut transversally to expose the inner portion of the scaffold and then samples were dehydrated at 4 °C through sequential exposure to a gradient of ethanol (in percentage: 50, 70, 80, 90, 95, 100), and then processed with a critical point dryer (Tousimis Autosamdri, USA), sputter coated with Au/Pd (Hummer VI Sputter Coater, Ladd Research Industries, USA) and analyzed by SEM (Supra55VP, Zeiss, Oberkochen, Germany) at 5 kV and 10 µA.

Control planar sponges were punched into cylinders via a 6 mm biopsy punch and left in PBS (1×) (ThermoFisher Scientific, Waltham, MA) for 30 min at 37 °C to equilibrate. Unconfined mechanical testing was performed on as‐prepared acellular planar sponges using a CellScale (UniVert CellScale biomaterial testing, Ontario, Canada) equipped with a 25 Newton load cell.

Tests were carried out using two parallel nonporous platens up to 80% strain. The compressive modulus of all samples was calculated from the slope of the initial linear region (<20% strain) of the stress–strain outputs (*N* = 6 for 3 independent experiments).^[^ ^50^ ^]^

The oxygen concentration profiles were measured using a PC‐controlled Microx TX3 oxygen meter (PreSens Precision Sensing GmbH, Rengensburg, Germany) equipped with a needle‐type housing fiber‐optic oxygen sensor (NTH‐PSt1‐L5‐TF‐NS40/0.8‐OIW, 140 µm fiber tapered to a 50 µm tip in the sulcus. The needle probe was mounted on a custom‐made micromanipulator capable of precisely positioning the measurement spot in the vertical direction. One complete turn of the screw knob resulted in 0.1 inch (2.5 mm) of travel. Oxygen concentration was measured weekly with a 500 µm step over the scaffold profile to monitor the oxygen profile within the sulcus over 6 weeks in culture, in comparison to acellular and planar controls (Figure S1, Supporting Information).

Cell distribution as well as tissue construct morphology and extracellular matrix distribution were assessed at 6 weeks in culture by CLSM and histological analyses. For imaging with CLSM, cells were stained with Calcein AM from LIVE/DEAD Viability/Cytotoxicity Kit (Life Technologies, Grand Island, NY) according to the manufacturer's instructions. Briefly, cells were incubated for 60 min and then washed 3 times in PBS and imaged using a CLSM with excitation at 488 nm and emission at 499–537 nm. For histological preparation, samples (*n* = 3) were cut into 2 parts to study cell distribution and washed in PBS (1×) and fixed in 10% neutral buffered formalin (Protocol, Fisher Scientific) overnight. Specimens were then processed, embedded in paraffin and cut in transverse sections of 7‐µm thickness. Histological sections were then deparaffinized with xylene, rehydrated through a series of graded ethanol, and stained with hematoxylin and eosin (H&E). Histological sections were analyzed with a light microscope (Keyence BZ‐X700) using a 10× and 20× objectives.

As part of the functional assessments, the gingival tissue was screened for epithelial barrier function with TEER. TEER measures nondestructively the integrity of tight junction dynamics in epithelial cell culture models, and it is a strong indicator of the integrity of the cellular barriers.^[^ ^51^ ^]^ EVOM^2^, Epithelial Volt/Ohm Meter was used with an EndOhm Chamber (World Precision Instrument) to measure TEER of the gingival construct after maturation in comparison to hGECs cultured for 7 days at air liquid interface (ALI) in a Transwell system (Corning, NY). TEER values were reported subtracting the contribution from the Transwell together silk scaffold with Collagen gel for the gingival construct and the Transwell chamber alone for the hGEC control.

Gingival constructs were washed with PBS (1×) (Thermo‐Fisher Scientific, Waltham, MA), fixed with 4% paraformaldehyde (Electron Microscopy Sciences, 157‐4) after teeth removal, and subsequently washed with additional PBS. Before permeabilization, the pockets were cut transversally to be able to image the epithelium and then cells were permeabilized with 0.25% Triton X‐100 (Sigma, T8787), followed by incubation in blocking buffer containing 1% bovine serum albumin (Sigma, A4503) and 10% horse serum at room temperature (Invitrogen). Primary antibodies against Ki67 (Abcam, ab15580, dilution 1:100), E‐cadherin (Abcam, ab1416, dilution 1:50) were added and incubated overnight at 4 °C, followed by multiple PBS washes. Constructs were then incubated with secondary fluorescent antibodies (Life Technologies) at room temperature and washed with PBS. Microscopy was performed with a Keyence BZ‐X700 microscope to image samples under 10× magnification obtained at 470–510 nm excitation over an emission range of 525–575 nm for green fluorescent protein and 560–600 nm excitation over an emission range of 630–705 nm for Texas Red (Keyence, Elmwood Park, NJ).

### Human Host‐Microbiome Interactions

The ability of the anatomical gingival tissue model to maintain and support the organization of complex human oral microbiome in vitro was validated by inoculating pooled plaque samples (*n* = 5) from healthy patients and cultured for 24 h. Human subgingival plaque and saliva samples were collected from donors with healthy periodontal tissues at the Center for Clinical and Translational Research at the Forsyth Institute, Cambridge (MA). Six plaque samples were combined and inoculated into the gingival sulcus in 3 µL aliquots. The microbiome was inoculated on the 7th day after seeding, to allow the construct to reach maturity, and then cultured for 24 h. The total duration of the experiment was 8 days. Saliva samples were pooled, then sterile filtered and combined with co‐culture medium at a 1:4 ratio. All methods were carried out in accordance with a protocol approved by the Institutional Review Board of Forsyth (Protocol No. FIRB# 18‐06), of Tufts University (Protocol No. IRB – 12860) and of University Massachusetts Lowell (Protocol No. IRB – 20‐090). All subjects signed FIRB approved informed consent prior to sampling.

### Human Host‐Microbiome Interactions Characterizations: Microbiome

SYTO 9 (Thermo‐Fisher Scientific, Waltham, MA) was used to fluorescently stain nucleic acid of the human oral microbiome pooled samples to investigate viability at 24 h in the anatomical gingival tissue model in comparison to the planar construct. Before inoculation, the pooled samples were incubated for 15 min in a PBS (1×) solution of 20 µm SYTO 9, then spun, washed and resuspended in culture medium for seeding. After multiple PBS washes to reduce background staining, samples were then imaged at 24h post inoculation using a CLSM with excitation at 488 nm and emission at 499–537 nm.

### 16S rRNA Sequencing Analysis

Samples collected at 24 h were processed for DNA isolation using the Epicentre MasterPure Gram Positive DNA Purification Kit (Lucigen, Middleton, WI, USA) (*N* = 5). Briefly, samples were resuspended in TE buffer and incubated over night at 37 °C after addition of the Ready‐Lyse lysozyme solution. Then, GP Lysis solution was added, and the samples were vortexed for 1 min followed by addition of Proteinase K and incubated at 65 °C for 15 min. Protein and RNA were then removed using MPC Protein Precipitation Reagent and RnaseA respectively. Lastly, DNA was precipitated with isopropanol (Sigma Aldrich, St. Louis, MO, USA), washed 2 times with ethanol 70% (Sigma Aldrich, St. Louis, MO, USA) and finally resuspended in water.

Bacterial 16S rRNA gene targeted amplicon sequencing was performed using a custom dual‐index protocol.^[^ ^52^ ^]^ The custom 16S primers used amplified the V1–V3 region of the 16S rRNA gene and were designed to provide the best coverage of the 16S gene while maintaining high sensitivity. The sample libraries were prepared using a 22 cycle PCR reaction to reduce chimera formation. The final PCR products were purified using Ampure XP beads (Beckman Coulter, Brea, CA, USA), pooled in equal amounts, and gel purified using the QIAGEN MinElute Gel Extraction Kit (QIAGEN, Hilden, Germany). Purified, pooled libraries were quantified using the NEBNext Library Quant Kit (New England Biolabs, Ipswich, MA, USA) for Illumina.

Final libraries were sequenced on Illumina MiSeq with a v2 reagent kit (Illumina, San Diego, CA, USA) (500 cycles) at the Human Oral Microbe Identification using Next Generation Sequencing core at the Forsyth Institute. The sequencing was performed at a 10pm loading concentration with >20% PhiX spike‐in. For analysis, the DADA2 R package^[^ ^53^ ^]^ was used to identify and quantify amplicon sequencing reads on the fastq files obtained after demultiplexing with the Illumina MiSeq software. Briefly, reads were trimmed and filtered to remove sequences with low quality. Quality of the trimmed and filtered reads was assessed using FastQC.^[^ ^54^ ^]^ Samples with read count smaller than 1000 reads per sample were excluded in the analysis. Results of FastQC were compiled using MultiQC.^[^ ^55^ ^]^ The trimmed and filtered reads were then processed through the denoising, concatenating read1 and read2 with a 10N spacer, and chimera removal steps of DADA2 to identify and quantify true amplicon sequence variants (ASV) present in the sample. Taxonomy of the identified amplicon sequence variants (ASV) was assigned using the RDP classifier algorithm^[^ ^56^ ^]^ implemented in the DADA2 package with a training dataset developed at the Forsyth Institute and based on the eHOMD^[^ ^57^ ^]^ generating a table with relative abundance of OTUs.

To calculate alpha diversity (Shannon index) and richness in the samples (Chao‐1), the OTUs with specie frequency from all samples were imported into the free software Past3.2 (https://folk.uio.no/ohammer/past/). To analyze changes in relative abundance and principal component analysis (PCA), normalized out tables for human plaque at time 0 and anatomical samples at 24 h, were imported into the open‐source software STAMP v2.1.3.^[^ ^58^ ^]^

### Human Host‐Microbiome Interactions Characterizations: Host

Relative DNA amount (host) was quantified using Quant‐iT picoGreen dsDNA assay kit (Thermo‐Fisher Scientific, Waltham, MA). Upon collection, anatomical and control sponges were stored at −80C before processing. Anatomical sponges seeded with microbiome were analyzed upon retrieval. Sponges were lysed in 500 µL of 0.05% Triton‐X (Sigma‐Aldrich, St. Louis, MO) for 30 min and then sonicated for 10 s using an ultrasonic bath sonicator. Both assays were then conducted according to the manufacturer's protocol by diluting the sample 1:5 (anatomical sponges) and 1:10 (cylindrical sponges) in TE buffer.

Epithelium exudates were collected from the periodontal pocket in the inoculated anatomical models in comparison to non‐inoculated samples at 24 h. Simultaneous analysis of multiple cytokine and chemokine biomarkers for inflammation (Granulocyte‐macrophage colony‐stimulating factor (GM‐CSF), Interferon gamma (IFN‐*γ*), Interleukin 10 (IL‐10), Interleukin 12p40 (IL‐12p40), Interleukin 17A (IL‐17A), Interleukin 1 Receptor antagonist (IL‐1RA), Interleukin 1 alpha (IL‐1*α*), Interleukin 1 beta (IL‐1*β*), Interleukin 2 (IL‐2), Interleukin 4 (IL‐4), Interleukin 6 (IL‐6), Interleukin 8 (IL‐8), Monocyte Chemoattractant Protein 1 (MCP‐1), Tumor Necrosis factor alpha [TNF‐*α*]) was carried out with Human Cytokine/Chemokine Magnetic Bead Panel (MilliPlex MAP, Millipore, Sigma). Median fluorescence intensity of each analyte was read using a MAGPIX system (Luminex). Concentrations of proteins of interest were calculated using the median fluorescence intensity and the standard curve of each analyte, as previously described.^[^ ^59^ ^]^ Resulting protein concentrations from multiplex analyses were normalized by their respective sample DNA content.

Two‐ sample Student’ *t*‐test, one‐way ANOVA and Bonferroni and Tukey post hoc tests post hoc test for mean comparisons, with a significance level of *p* < 0.05 were used to assess statistical significance in the 3D humanized model of the periodontal gingival pocket (OriginPro 2022b, OriginLab Corporation, Northampton, MA, USA). For statistical analysis and dynamic identification of microbiome differences by relative abundance between the groups, STAMP v2.1.3 software was used. A Welch's *t*‐test for unequal variances was used to assign *p*‐values to the OTUs when comparisons between two groups were done. Values of *p < 0.05, **p < 0.01, and ***p < 0.001 were considered statistically significant. Data reported in the manuscript are given as mean ± SD, and a minimum of *n* = 3 was used for all experiments performed.

## Conflict of Interest

The authors declare no conflict of interest.

## Supporting information

Supporting Information

## Data Availability

The data that support the findings of this study are available from the corresponding author upon reasonable request.

## References

[1] M. A. Nazir , Int. J. Health Sci. 2017, 11, 72. PMC5426403 28539867

[2] S. Kurgan , A. Kantarci , Periodontol 2000 2018, 76, 51. 29194785 10.1111/prd.12146

[3] G. Hajishengallis , R. J. Lamont , Eur. J. Immunol. 2014, 44, 328. 24338806 10.1002/eji.201344202 PMC3925422

[4] a) G. Hajishengallis , R. J. Lamont , Mol. Oral Microbiol. 2012, 27, 409; 23134607 10.1111/j.2041-1014.2012.00663.x PMC3653317

[5] a) N. Suzuki , M. Yoneda , T. Hirofuji , Int. J. Dent. 2013, 2013, 1; 10.1155/2013/587279 PMC3606728 23533413

[6] S. Bamashmous , G. A. Kotsakis , K. A. Kerns , B. G. Leroux , C. Zenobia , D. Chen , H. M. Trivedi , J. S. McLean , R. P. Darveau , Proc. Natl. Acad. Sci. USA. 2021, 118, e2012578118. 34193520 10.1073/pnas.2012578118 PMC8271746

[7] a) J. Marchesan , M. S. Girnary , L. Jing , M. Z. Miao , S. Zhang , L. Sun , T. Morelli , M. H. Schoenfisch , N. Inohara , S. Offenbacher , Y. Jiao , Nat. Protoc. 2018, 13, 2247; 30218100 10.1038/s41596-018-0035-4 PMC6773250

[8] C. Rojas , M. P. García , A. F. Polanco , L. González‐Osuna , A. Sierra‐Cristancho , S. Melgar‐Rodríguez , E. A. Cafferata , R. Vernal , Front. Immunol. 2021, 12, 663328. 34220811 10.3389/fimmu.2021.663328 PMC8248545

[9] a) J. L. Brown , W. Johnston , C. Delaney , R. Rajendran , J. Butcher , S. Khan , D. Bradshaw , G. Ramage , S. Culshaw , Sci. Rep. 2019, 9, 15779; 31673005 10.1038/s41598-019-52115-7 PMC6823452

[10] J. L. M. Welch , F. E. Dewhirst , G. G. Borisy , Annu. Rev. Microbiol. 2019, 73, 335. 31180804 10.1146/annurev-micro-090817-062503 PMC7153577

[11] a) J. M. Gosline , M. E. DeMont , M. W. Denny , Endeavour 1986, 10, 37;

[12] a) F. G. Omenetto , D. L. Kaplan , Science 2010, 329, 528; 20671180 10.1126/science.1188936 PMC3136811

[13] a) C. E. Ghezzi , L. Wang , I. Behlau , J. Rnjak‐Kovacina , S. Wang , M. H. Goldstein , J. Liu , J. K. Marchant , M. I. Rosenblatt , D. L. Kaplan , J. Appl. Biomater. Funct. Mater. 2016, 14, e266; 27230452 10.5301/jabfm.5000274

[14] a) G. H. Altman , F. Diaz , C. Jakuba , T. Calabro , R. L. Horan , J. Chen , H. Lu , J. Richmond , D. L. Kaplan , Biomaterials 2003, 24, 401; 12423595 10.1016/s0142-9612(02)00353-8

[15] M. Adelfio , C. E. Ghezzi , ACS Biomater. Sci. Eng. 2022, 10.1021/acsbiomaterials.1c01380. PMC9508280 35324141

[16] V. Nandakumar , S. Chittaranjan , V. M. Kurian , M. Doble , Polym. J. 2013, 45, 4613.

[17] B. Nugala , B. S. Kumar , S. Sahitya , P. M. Krishna , J. Conservative Dent. 2012, 15, 137. 10.4103/0972-0707.92599 PMC3284004 22368328

[18] A. L. Griffen , C. J. Beall , J. H. Campbell , N. D. Firestone , P. S. Kumar , Z. K. Yang , M. Podar , E. J. Leys , ISME J. 2012, 6, 12. 10.1038/ismej.2011.191 PMC3358035 22170420

[19] J. Rnjak‐Kovacina , L. S. Wray , K. A. Burke , T. Torregrosa , J. M. Golinski , W. Huang , D. L. Kaplan , ACS Biomater. Sci. Eng. 2015, 1, 1176. 10.1021/ab500149p PMC4426347 25984573

[20] L. Shang , D. Deng , J. K. Buskermolen , M. M. Janus , B. P. Krom , S. Roffel , T. Waaijman , C. van Loveren , W. Crielaard , S. Gibbs , Sci. Rep. 2018, 8, 260. 30375445 10.1038/s41598-018-34390-y PMC6207751

[21] C. Rahimi , B. Rahimi , D. Padova , S. A. Rooholghodos , D. R. Bienek , X. Luo , G. Kaufman , C. B. Raub , Biomicrofluidics 2018, 12, 054106. 30310527 10.1063/1.5048938 PMC6158033

[22] a) W. J. Loesche , J. P. Robinson , M. Flynn , J. L. Hudson , R. E. Duque , Infect. Immun. 1988, 56, 156; 3335401 10.1128/iai.56.1.156-160.1988 PMC259250

[23] R. N. Eskow , W. J. Loesche , Arch. Oral Biol. 1971, 16, 1127. 5293413 10.1016/0003-9969(71)90218-4

[24] A. Koller , A. Sapra , in StatPearls Publishing Copyright © 2022, StatPearls Publishing LLC, Treasure Island, FL 2022.

[25] K. B. Lagosz‐Cwik , A. Wielento , W. Lipska , M. Kantorowicz , D. Darczuk , T. Kaczmarzyk , S. Gibbs , J. Potempa , A. M. Grabiec , Sci. Rep. 2021, 11, 10770. 34031466 10.1038/s41598-021-90037-5 PMC8144196

[26] K. Chwalek , D. Sood , W. L. Cantley , J. D. White , M. Tang‐Schomer , D. L. Kaplan , J. Visualized Exp. 2015, 105, e52970. 10.3791/52970 PMC4692668 26555926

[27] S. Szymkowiak , N. Sandler , D. L. Kaplan , ACS Appl. Mater. Interfaces 2021, 13, 10768. 33621042 10.1021/acsami.1c00548

[28] a) G. Kasnak , D. Fteita , O. Jaatinen , E. Könönen , M. Tunali , M. Gürsoy , U. K. Gürsoy , Histochem. Cell Biol. 2019, 152, 63; 30767049 10.1007/s00418-019-01774-8

[29] R. A. Crabb , E. P. Chau , D. M. Decoteau , A. Hubel , Ann. Biomed. Eng. 2006, 34, 10768. 10.1007/s10439-006-9181-x 17016762

[30] a) S. Groeger , J. Meyle , Front. Immunol. 2019, 10, 63; 30837987 10.3389/fimmu.2019.00208 PMC6383680

[31] X. Sun , P. D. Kaufman , Chromosoma 2018, 127, 1615. 10.1007/s00412-018-0659-8 PMC5945335 29322240

[32] A. Dongari‐Bagtzoglou , H. Kashleva , Nat. Protoc. 2006, 1, 2012. 17487190 10.1038/nprot.2006.323 PMC2699620

[33] B. C. Dickinson , C. E. Moffatt , D. Hagerty , S. E. Whitmore , T. A. Brown , D. T. Graves , R. J. Lamont , Mol. Oral Microbiol. 2011, 26, 175. 21545698 10.1111/j.2041-1014.2011.00609.x PMC3248246

[34] C. E. Ghezzi , B. Marelli , N. Muja , N. Hirota , J. G. Martin , J. E. Barralet , A. Alessandrino , G. Freddi , S. N. Nazhat , Biotechnol. J. 2011, 6, 2012. 10.1002/biot.201100127 21751393

[35] T. Tuganbaev , K. Yoshida , K. Honda , Science 2022, 376, 210. 35617380 10.1126/science.abn1890

[36] A. Sarkar , F. Xu , S. Lee , Adv. Colloid Interface Sci. 2019, 273, 1198. 10.1016/j.cis.2019.102034 31518820

[37] J. L. M. Welch , B. J. Rossetti , C. W. Rieken , F. E. Dewhirst , G. G. Borisy , Proc. Natl. Acad. Sci. U. S. A. 2016, 113, 934. 10.1073/pnas.1522149113 PMC4760785 26811460

[38] L. Abusleme , A. K. Dupuy , N. Dutzan , N. Silva , J. A. Burleson , L. D. Strausbaugh , J. Gamonal , P. I. Diaz , ISME J. 2013, 7, 102034. 10.1038/ismej.2012.174 PMC3635234 23303375

[39] a) S. Jalili‐Firoozinezhad , F. S. Gazzaniga , E. L. Calamari , D. M. Camacho , C. W. Fadel , A. Bein , B. Swenor , B. Nestor , M. J. Cronce , A. Tovaglieri , O. Levy , K. E. Gregory , D. T. Breault , J. M. S. Cabral , D. L. Kasper , R. Novak , D. E. Ingber , Nat. Biomed. Eng. 2019, 3, 520; 31086325 10.1038/s41551-019-0397-0 PMC6658209

[40] Z. M. Burcham , N. L. Garneau , S. S. Comstock , R. M. Tucker , R. Knight , J. L. Metcalf , A. Miranda , B. Reinhart , D. Meyers , D. Woltkamp , E. Boxer , J. Hutchens , K. Kim , M. Archer , M. McAteer , P. Huss , R. Defonseka , S. Stahle , S. Babu , T. Nuessle , V. Schowinsky , W. Covert , W. Truman , W. Reusser , Genetics of Taste Lab Citizen Scientists , Sci. Rep. 2020, 10, 1016. 32034250 10.1038/s41598-020-59016-0 PMC7005749

[41] P. D. Marsh , Microb. Ecol. Health Dis. 2000, 12, 130.

[42] P. N. Deo , R. Deshmukh , J. Oral Maxillofac. Pathol. 2019, 23, 122. 10.4103/jomfp.JOMFP_304_18 PMC6503789 31110428

[43] a) C. A. Brennan , W. S. Garrett , Nat. Rev. Microbiol. 2019, 17, 156; 30546113 10.1038/s41579-018-0129-6 PMC6589823

[44] a) A. M. L. Pedersen , D. Belstrøm , J. Dent. 2019, 80, S3;30696553

[45] a) B. J. Paster , S. K. Boches , J. L. Galvin , R. E. Ericson , C. N. Lau , V. A. Levanos , A. Sahasrabudhe , F. E. Dewhirst , J. Bacteriol. 2001, 183, 156; 10.1128/JB.183.12.3770-3783.2001 PMC95255 11371542

[46] a) D. A. Scott , J. Krauss , Front. Oral Biol. 2012, 15, S3; 10.1159/000329672 PMC3335266 22142957

[47] D. H. Thunell , K. D. Tymkiw , G. K. Johnson , S. Joly , K. K. Burnell , J. E. Cavanaugh , K. A. Brogden , J. M. Guthmiller , J. Periodontal Res. 2010, 45, 148. 19602112 10.1111/j.1600-0765.2009.01204.x PMC5405696

[48] N. Arias‐Bujanda , A. Regueira‐Iglesias , M. Alonso‐Sampedro , M. M. González‐Peteiro , A. Mira , C. Balsa‐Castro , I. Tomás , Sci. Rep. 2018, 8, 745. 30573746 10.1038/s41598-018-35920-4 PMC6301951

[49] D. N. Rockwood , R. C. Preda , T. Yucel , X. Wang , M. L. Lovett , D. L. Kaplan , Nat. Protoc. 2011, 6, 148. 10.1038/nprot.2011.379 PMC3808976 21959241

[50] F. Chicatun , C. E. Pedraza , C. E. Ghezzi , B. Marelli , M. T. Kaartinen , M. D. McKee , S. N. Nazhat , Biomacromolecules 2011, 12, 2946. 21661759 10.1021/bm200528z

[51] B. Srinivasan , A. R. Kolli , M. B. Esch , H. E. Abaci , M. L. Shuler , J. J. Hickman , J. Lab. Autom. 2015, 20, 1612. 10.1177/2211068214561025 PMC4652793 25586998

[52] J. J. Kozich , S. L. Westcott , N. T. Baxter , S. K. Highlander , P. D. Schloss , Appl. Environ. Microbiol. 2013, 79, 2946. 10.1128/AEM.01043-13 PMC3753973 23793624

[53] B. J. Callahan , P. J. McMurdie , M. J. Rosen , A. W. Han , A. J. A. Johnson , S. P. Holmes , Nat. Methods 2016, 13, 107. 27214047 10.1038/nmeth.3869 PMC4927377

[54] S. Andrews , A Quality Control Tool for High Throughput Sequence Data, www.bioinformatics.babraham.ac.uk/projects/fastqc/ (accessed: May 2020).

[55] P. Ewels , M. Magnusson , S. Lundin , M. Käller , Bioinformatics 2016, 32, 581. 10.1093/bioinformatics/btw354 PMC5039924 27312411

[56] Q. Wang , G. M. Garrity , J. M. Tiedje , J. R. Cole , Appl. Environ. Microbiol. 2007, 73, 5261. 17586664 10.1128/AEM.00062-07 PMC1950982

[57] I. F. Escapa , T. Chen , Y. Huang , P. Gajare , F. E. Dewhirst , K. P. Lemon , mSystems 2018, 3, 3047. 10.1128/mSystems.00187-18 PMC6280432 30534599

[58] D. H. Parks , G. W. Tyson , P. Hugenholtz , R. G. Beiko , Bioinformatics 2014, 30, 5261. 10.1093/bioinformatics/btu494 PMC4609014 25061070

[59] P. Diaz‐Rodriguez , H. Chen , J. D. Erndt‐Marino , F. Liu , F. Totsingan , R. A. Gross , M. S. Hahn , ACS Appl. Bio Mater. 2019, 2, 601. 10.1021/acsabm.8b00799 35016323

[60] B. C. Dickinson , C. E. Moffatt , D. Hagerty , S. E. Whitmore , T. A. Brown , D. T. Graves , R. J. Lamont , Mol. Oral Microbiol. 2011, 26, 3123. 10.1111/j.2041-1014.2011.00609.x PMC3248246 21545698

