# Supplementary material for: Three‐Dimensional Humanized Model of the Periodontal Gingival Pocket to Study Oral Microbiome
Source: Adv Sci (Weinh). 2023 Feb 24;10(12):2205473. doi: 10.1002/advs.202205473 (PMC10131835; doi:10.1002/advs.202205473)
Supplement: Supplementary file 1 — Supporting Information [file ADVS-10-2205473-s001.pdf]

ADVANCED SCIENCE
Open Access

Supporting Information

for *Adv. Sci.*, DOI 10.1002/advs.202205473

Three-Dimensional Humanized Model of the Periodontal Gingival Pocket to Study Oral Microbiome

*Miryam Adelfio, Zaira Martin-Moldes, Joshua Erndt-Marino, Lorenzo Tozzi, Margaret J. Duncan, Hatice Hasturk, David L. Kaplan and Chiara E. Ghezzi**

Supporting Information

Title: ***Three-dimensional humanized model of the periodontal gingival pocket to study oral microbiome***

Miryam Adelfio, Zaira Martin-Moldes, Joshua Erndt-Marino, Lorenzo Tozzi, Margaret J. Duncan, Hatice Hasturk, David L. Kaplan, and Chiara E. Ghezzi

Corresponding Author: Chiara E. Ghezzi, Chiara_Ghezzi@uml.edu

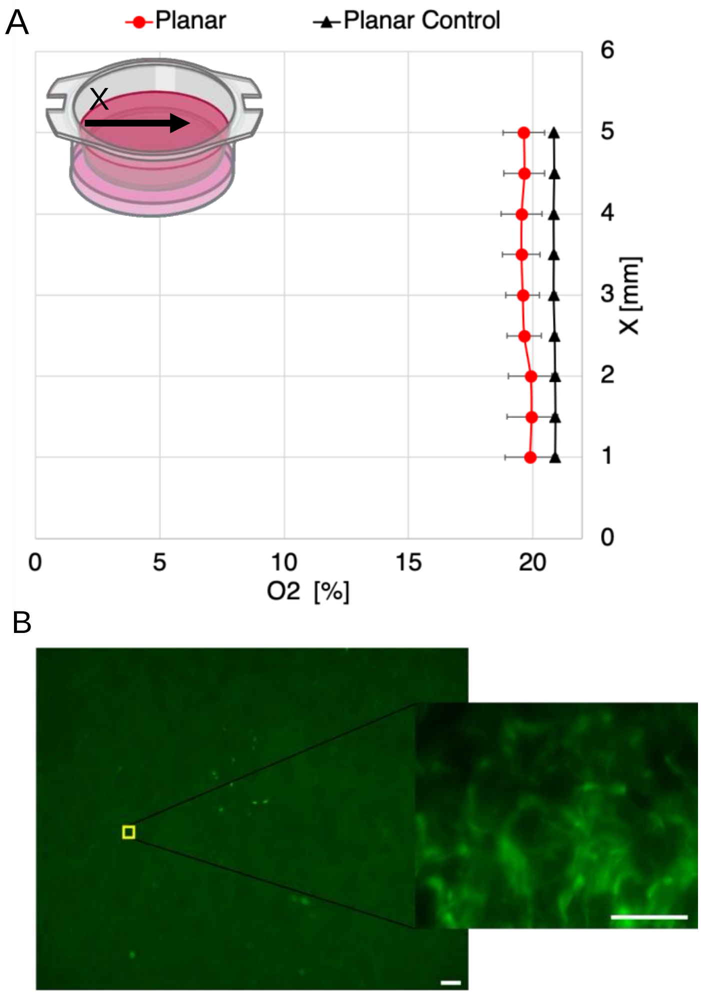

Supplementary Figure 1- Planar tissue control characterization. A) Spatial oxygen profile of the cellularized planar control (red line) in comparison to acellular plain scaffold (black line). B) Microbiome viability assessment at 24 h in the planar control scaffold. Maximum intensity projection of CLSM analysis of Syto-9 positively stained human oral microbiome. Scale bars = 100 µm.

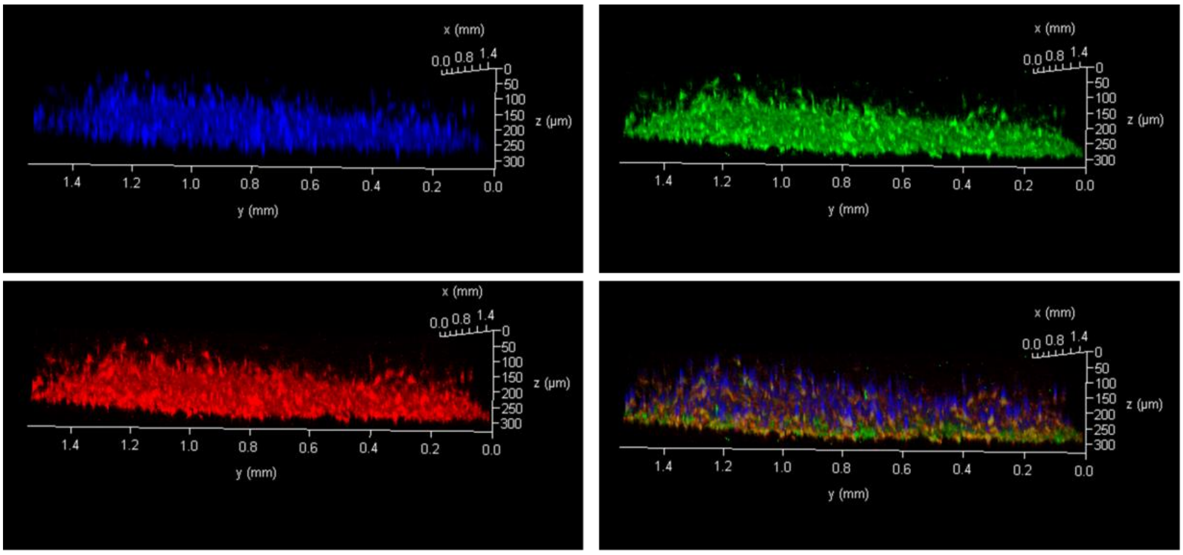

Supplementary Figure 2: CLSM 3D rendering of the epithelium in the anatomical model of DAPI (blue), Ki67 (red), E-Cadherin (green) and merge.

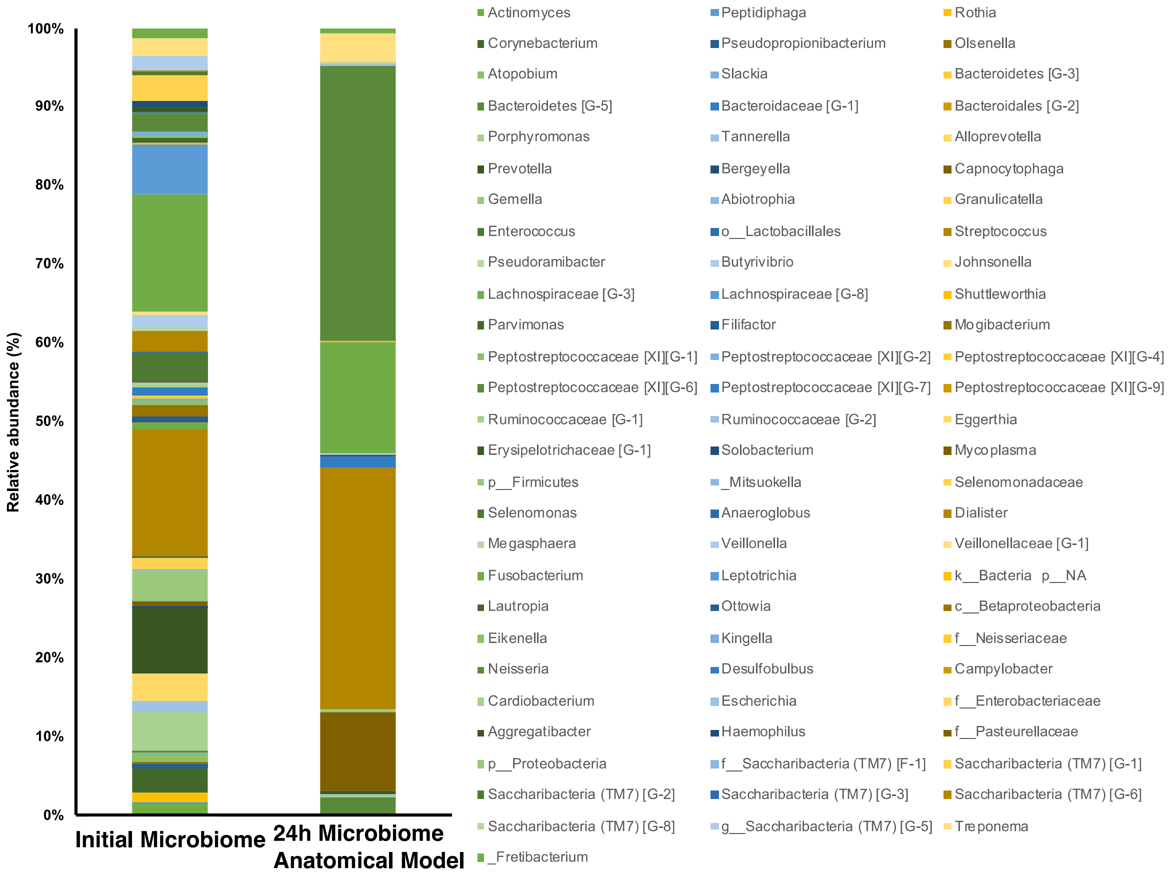

Supplementary Figure 3: Relative abundance distribution of main genera detected in human plaque before incubation (Initial Microbiome) samples and samples collected after 1 day incubation in the anatomical model (24h Microbiome Anatomical Model).

Supplementary Table 1: Statistical differences in changes of relative abundance at the level of specie. The table summarizes the Fold change and p-value with a 95% confidence.

Specie	Log2 fold change	log10 p vaue
g_Neisseria	6,83	1,4

Neisseria subflava	6,01	2,06
Streptococcus constellatus	5,30	4,03
o_Lactobacillales	4,69	2,55
g_Gemella	4,46	1,65
Solobacterium moorei	3,64	2,31
Fusobacterium nucleatum subsp. polymorphum	2,59	2,1
Capnocytophaga sputigena	2,30	2,37
Fusobacterium periodonticum	-1,43	2,34
Streptococcus cristatus	-2,48	1,51
k_Bacteria	-2,58	3,04
Porphyromonas catoniae	-2,87	3,35
Eikenella corrodens	-3,08	1,75
Dialister pneumosintes	-3,11	1,36
Prevotella micans	-3,18	1,78
Fusobacterium nucleatum subsp.animalis	-3,30	1,96
Streptococcus infanti	-3,43	2,68
Mycoplasma salivarium	-3,44	2,21
Aggregatibacter sp.HMT513	-4,15	1,41
Prevotella sp.HMT317	-4,30	1,31

Streptococcus gordonii	-4,30	1,96
Abiotrophia defectiva	-4,38	2,26
Alloprevotella sp.HMT473	-4,54	1,52
Fusobacterium nucleatum subsp.vincentii	-4,60	2,69
Filifactor alocis	-4,61	1,77
Neisseria macacae:flava:mucosa:sicca	-4,97	1,43
Gemella sanguinis	-5,20	1,74
Alloprevotella tannerae	-6,30	1,45
f_Neisseriaceae	-6,50	1,96
Saccharibacteria (TM7) [G-1] bacterium_HMT952	-6,56	2,02
Saccharibacteria (TM7) [G-1] bacterium_HMT349	-6,57	2,06
Prevotella micans	-6,62	1,71
Prevotella maculosa	-7,11	1,47
Peptostreptococcaceae yurii subspp. Yurii & margaretiae	-7,15	1,51
Prevotella intermedia	-7,17	1,34
Gemella bergeri	-7,44	4,13
Saccharibacteria (TM7) [G-5] bacterium_HMT356	-7,46	1,55
Prevotella nigrescens	-7,59	1,98
Aggregatibacter sp.HMT458	-7,64	1,65

g_Selenomonas	-7,77	1,91
Prevotella nanceiensis	-7,81	1,74
Streptococcus intermedius	-8,18	1,34
Lachnospiraceae [G-3] bacterium_HMT100	-8,22	2,89
Saccharibacteria (TM7) [G-1] bacterium_HMT346	-9,09	1,94
Tannerella forsythia	-9,41	1,46
Selenomonas sputigena	-9,80	2,18
Fusobacterium nucleatum subsp.vincentii:naviforme	-9,95	2,25
Dialister invisus	-10,04	3,88
Fusobacterium naviforme	-11,66	2,72
Porphyromonas endodontalis	-11,88	1,93
